# Implications of respiratory motion for small animal image-guided radiotherapy

**DOI:** 10.1259/bjr.20160482

**Published:** 2017-01

**Authors:** Mark A Hill, Borivoj Vojnovic

**Affiliations:** Cancer Research UK/Medical Research Council Oxford Institute for Radiation Oncology, Gray Laboratories, University of Oxford, Old Road Campus Research Building, Roosevelt Drive, Oxford OX3 7DQ, UK

## Abstract

Image-guided small animal irradiators have the potential to make a significant impact on facilitating the translation of radiobiological research into the clinic. To fully exploit the improved precision in dose delivery to the target/tumour while minimizing dose to surrounding tissues, minimal positional error in the target is required. However, for many sites within the thorax and abdomen, respiratory motion may be a critical factor in limiting the accuracy of beam delivery and until now, very little attention has been paid to the impact and management of this motion. We report on the implications of respiratory motion with respect to the negative impact of delivered dose distributions and their assessment, ways being developed to effectively manage this motion, so that beam delivery only occurs during the stationary resting phase of the breathing cycle, and comment on the need to effectively integrate these developments into the software used to plan and control beam delivery. Altogether, the implementation of respiratory-gated imaging and beam delivery will lead to significant improvements in the precision in dose delivery and constitutes an important development for preclinical radiotherapy studies.

## CURRENT STATUS

The recent development of image-guided small animal irradiators has the potential to make a significant impact on facilitating the translation of radiobiological research into the clinic.^1,2^ These devices are particularly useful when targeting and treating orthotopic tumours at internal sites, with imaging at treatment time to determine the size and location of the tumour and precise treatment with tightly collimated beams. Associated treatment-planning software also enables complex treatments to be planned and the three-dimensional dose distribution to be calculated, through either the use of multiple beams or delivering beams using dynamic arcs in conjunction with gantry or couch rotation. In order to fully exploit this improved precision, minimal positional error in the target is required. While this can relatively easily be achieved in anatomic sites with minimal movement during treatment (such as the brain), organs within the thorax and abdominal regions will be affected by breathing to varying degrees depending on their location. As is the case with human radiotherapy, there is an increasing need to take into account organ motion during beam delivery in order to achieve the required precision in dose delivery to the target while minimizing dose to the surrounding tissues, especially critical organs, and so achieving the highest therapeutic index. Until now, very little attention has been paid to respiratory motion, its negative impact on delivered dose distributions or ways to address this. However, this was the topic of several presentations at the Third Symposium on Precision Image-guided Small Animal Radiotherapy held recently from 21–23 March 2016 in Ghent, Belgium.

## IMPLICATIONS OF RESPIRATORY MOTION

For many sites within the thorax and abdomen, respiratory motion may be a critical factor in limiting the accuracy of beam delivery. The degree of motion is dependent on position and can be up to the order of 5 mm in places in mice and is likely to be greater in rats. The movement of the tumour in and out of the treatment field will typically lead to an increase in the heterogeneity of the dose across the tumour, with an associated reduction in the average dose. Not only does the target/tumour move during the breathing cycle, but also the relative position of organs and therefore the dose to these organs. Unlike humans, the breaths taken by an anesthetized rodent tend to be pulsatile in nature, consisting of short gasps separated by an extended resting phase. As a result, the effects of respiratory motion are likely to become more significant with increasing breathing rates, owing to a decrease in the fraction of time during the breathing cycle taken by the stationary resting phase. One option would be to increase the overall dose to the tumour to ensure that all parts of the tumour receive the required dose, but this would also increase dose to the surrounding normal tissue. In addition, if the extent of the motion is known, then the field size could be increased to encompass tumour movement. However, this will result in an increase in the volume of the irradiated normal tissue and associated dose, which may have implications on the effectiveness and consequences of the treatment protocol. In either case, current treatment-planning software will typically use a stationary image and the calculated dose distributions to the tumour and surrounding organs will not take this motion into account. Therefore, in order to accurately assess dose distributions, it is important to assess this movement as a function of time, using time-weighted images to determine the dose distribution through various stages of the cycle. Using this approach with the four-dimensional MOBY phantom (Duke University, Durham, MC), van der Heyden et al^3^ from Maastro (Maastrict, Netherlands) investigated the impact of breathing motion on mouse lung tumour irradiation and predicted an 11% reduction in mean dose to a tumour located near the diaphragm, compared with planned dose (without motion). However, in order to achieve maximum accuracy when using image-guided irradiators for moving targets, X-ray beam delivery needs to account for respiratory motion.

Four-dimensional cone-beam CT (CBCT) imaging is not presently available on current-generation image-guided small animal irradiators and would be difficult to perform without a significant increase in the imaging dose. Other imaging modalities thus need to be exploited to assess movement. If such imaging is performed on independent devices, then great care must be taken to minimize overall animal movement and associated internal organ movement during transport to the irradiator, prior to co-registration with on-board CBCT images.

## MANAGEMENT OF RESPIRATORY MOTION

Optimal beam delivery requires effective management of respirator motion, so that beam delivery only occurs during the stationary resting phase of the breathing cycle. One way to address the problem would be to intubate and ventilate the rodent in order to externally control the breathing cycle, so that breaths occurred only while X-rays were off. However, this is a highly skilled procedure in view of the small size of the trachea and care is needed to avoid damage to the trachea.^4^ Alternatively, the motion can be monitored to provide an external signal with which the irradiation can be synchronized. This is generally performed indirectly by following surface motion with the actual tumour/target position derived by correlating output with previous imaging. Movement is dependent on depth of anaesthesia and follows, in general, a pulsatile pattern. While it would be beneficial to minimize the depth of anaesthesia due to potential effects on physiology,^5^ this would reduce the fraction of time spent in the resting phase and increase the chance of a gross movement of the animal so the target and body position no longer agrees with that used for planning. This in turn may lead to major issues with beam delivery. It is important that respiratory-gated delivery be able to deal with changes in the period between breaths and minimize the impact of an unexpectedly early breath. On the other hand, it is important that the on/off duty cycle of the beam is not so low that it leads to a decrease in the biological effective dose owing to ongoing repair during delivery.^6^

## RESPIRATORY-GATED BEAM DELIVERY

To address this issue, the University of Oxford have developed a gated treatment head assembly for the Xstrahl's SARRP irradiator (Xstrahl Ltd., Camberley, UK), which incorporates a fast rotating X-ray shutter, along with optical breathing monitoring technology and associated adaptive gating control (study in preparation).^7,8^ The system can cope with all realistic breathing rates, as well as irregularities in the breathing period. The fast response time of the shutter (<20 ms latency) means that the X-rays can be quickly turned off, should a breath be detected earlier than expected, thereby restricting delivery to breathing cycle periods when tissue movement is minimal. The successful application of this technology has been demonstrated *in vivo* on an anaesthetized mouse for breathing rates from 40 to 100 bpm, by comparing movies obtained using planar X-ray imaging and dose profiles obtained using a collimated beam with and without respiratory gating. The optical breathing monitor can also be used to obtain respiratory-gated MR images for use in treatment planning.

Respiratory gating is being addressed using a linear shutter (120-ms open/close time) in conjunction with a commercial pressure balloon-based system at the Advanced Resource Center for Hadrontherapy in Europe in France.^8,9^ A dynamic phantom developed by this group suggests that gating can be used to successfully compensate for a sinusoidal motion and minimize image blur for positron emission tomography and CT imaging.

An alternative to gated beam delivery enabling continuous beam delivery would be to employ beam tracking rather than gating. However, this is a tough engineering challenge on current commercial platforms owing to the significant power required to move heavy items with appropriate acceleration/deceleration. It would be easier to stop the rotation/translation of the animal, but care would be needed to ensure accuracy. Since the compliance (low stiffness) of most small animals tends to be high, the challenge will be to ensure that the required high accelerations and decelerations do not affect the position of the internal target.

### Treatment planning

Treatment planning for respiratory-gated beam delivery should ideally be performed with images also taken during the resting phase of the breathing cycle. This is not always possible; the on-board CBCT image obtained using these platforms will typically include image-blurring artefacts as a result of respiratory motion and will typically represent average organ positions. Fortunately, for anesthetized rodents, the majority of time over the breath cycle is spent in the resting phase, punctuated by a short gasp, but this of course will reduce at high breathing rates.

Planning the delivery of single or multiple stationary respiratory-gated beams is essentially the same as for non-gated beams; however, the calculated delivery times now refer to “beam-on” times rather than actual time. However, planning and delivery of dynamic arc treatments (by rotating either the gantry with the animal fixed or the animal with the gantry fixed) is more problematic. The current planning software will assume continuous beam delivery, while in practice, beam delivery will be discontinuous. To a first approximation, this can be accounted for by using the on/off duty cycle, to predict the expected time for dose delivery and reducing the speed of rotation so that the time the motion is due to finish corresponds to the expected time to deliver the required dose for an ungated beam. However, to determine the actual dose delivered would require the treatment-planning software to calculate the dose distributions using the actual beam delivery time course. The mass of the gantry with tube would make it difficult to stop the gantry rotation during the beam-off periods and would also significantly affect the accuracy of beam delivery. As mentioned previously, interrupting the rotation/translation of the animal could also be investigated. Alternatively, rather than using a continuous arc, the movement could be split into a fixed number of known beam positions rather than a continuous arc at the cost of overall time for delivery. This also gives the potential to make improvements in dose distribution by changing dose delivery as a function of position. Another option would be to slightly improve the uniformity by averaging dose delivery over multiple rather than a single pass, but this may not always be practical, without reducing the dose rate and significantly increasing delivery time.

There has recently been significant effort in developing “dose-painting” approaches, using combinations of small fields to produce irregular-shaped dose distributions to a target volume or non-uniform dose distributions.^10–13^ These techniques require delivery with very high precision and therefore, respiratory motion can result in significant deterioration in the delivered dose distributions and would therefore significantly benefit from respiratory-gated beam delivery.

## SUMMARY

Altogether, the implementation of respiratory-gated imaging and beam delivery will lead to significant improvements in the precision in dose delivery and constitutes an important development for preclinical radiotherapy studies in the thorax and abdomen, especially once fully integrated with the software used to plan and control beam delivery.

## FUNDING

Funding from Cancer Research UK (programme grants C5255/A15935, C5255/A12678) and the Medical Research Council Strategic Partnership Funding (MRC-PC-12001) for the CRUK/MRC Oxford Institute for Radiation Oncology is gratefully acknowledged.
